# Propionate Fermentative Genes of the Gut Microbiome Decrease in Inflammatory Bowel Disease

**DOI:** 10.3390/jcm10102176

**Published:** 2021-05-18

**Authors:** Juan Manuel Medina, Raúl Fernández-López, Javier Crespo, Fernando de la Cruz

**Affiliations:** 1 Instituto de Biomedicina y Biotecnología de Cantabria (IBBTEC), 39011 Santander, Spain; jmedina@idival.org (J.M.M.); raul.fernandez@unican.es (R.F.-L.); 2 Clinical and Translational Digestive Research Group, Gastroenterology and Hepatology Department, IDIVAL, Marqués de Valdecilla University Hospital, 39008 Santander, Spain; javier.crespo@scsalud.es

**Keywords:** short chain fatty acids, microbiota, Crohn’s disease, metagenomics

## Abstract

Changes in the gut microbiome have been associated with inflammatory bowel disease. A protective role of short chain fatty acids produced by the gut microbiota has been suggested as a causal mechanism. Nevertheless, multi-omic analyses have failed to identify a clear link between changes in specific taxa and disease states. Recently, metagenomic analyses unveiled that gut bacterial species have a previously unappreciated genomic diversity, implying that a geno-centric approach may be better suited to identifying the mechanisms involved. Here, we quantify the abundance of terminal genes in propionate-producing fermentative pathways in the microbiome of a large cohort of healthy subjects and patients with inflammatory bowel disease. The results show that propionate kinases responsible for propionate production in the gut are depleted in patients with Crohn’s disease. Our results also indicate that changes in overall species abundances do not necessarily correlate with changes in the abundances of metabolic genes, suggesting that these genes are not part of the core genome. This, in turn, suggests that changes in strain composition may be as important as changes in species abundance in alterations of the gut microbiome associated with pathological conditions.

## 1. Introduction

Many pathological conditions of the gut are linked to changes in the composition of the gut microbiome (GM). Microbes in the gut produce metabolites essential for enterocyte function. Many of these metabolites regulate the integrity of the intestinal barrier, and some were shown to exert an immunomodulatory role [1]. Because of this interplay between the GM metabolism and intestinal function, it has been proposed that changes in the GM may be involved in the genesis and/or evolution of several intestinal diseases. One of the most widely studied associations between GM changes and gut pathology is inflammatory bowel disease (IBD). IBD is a gastrointestinal disorder characterized by a chronic inflammation of the gastrointestinal tract, associated in some cases with extraintestinal signs of systemic inflammation. IBD comprises two clinical manifestations: Crohn´s disease (CD) and ulcerative colitis (UC). Studies have repeatedly shown that the GM composition of IBD patients differs from healthy subjects [2]. However, the species involved and the magnitude and sign of the changes observed are highly variable, and most taxa appear to increase their numbers in some studies, but decreasing in others [3]. This variability complicates the elucidation of a causal link between GM alterations and the onset and progress of IBD. However, in recent years, substantial evidence has accumulated on the potential role of short chain fatty acids (SCFAs) as possible mediators between GM alterations and IBD [4].

SCFAs such as acetate, propionate, and butyrate are produced by the GM from the anaerobic fermentation of carbohydrates and amino acids present in dietary fiber [5]. SCFAs are important nutrients for enterocytes, which use them as a primary source of energy. They also promote the development of regulatory T lymphocytes, which modulate tissue inflammation and cytokine production [6]. These functional roles may have a clinical interaction since dietary information from IBD patients correlated higher fiber intake with a lower risk of CD [7]. It was proposed that SCFA production from dietary fiber by the GM may be implicated in a protective effect on the intestinal epithelium [8]; however, the mechanisms of such an effect are still unclear. Whereas studies in germ-free mice showed that SCFAs promoted T regulatory cell development [9] and protected against T-cell-mediated colitis [10], multi-omic studies in humans have produced less conclusive results. In IBD patients, a depletion in certain butyrate-producing bacteria such as *Faecalibacterium* or *Roseburia* has been observed [11,12,13]. However, these studies failed to reveal a univocal relationship between changes in GM composition and SCFA concentrations.

One possibility for this discrepancy is that changes in overall species composition, as identified by 16S metataxonomic studies, do not directly correlate with variations in the metabolic capabilities of the GM. Recent studies showed that bacterial species within the human gut exhibit substantial genomic variation, each exhibiting up to hundreds of genomically different species [14]. If taxonomic labels correlate poorly with metabolic capability, it is possible that pathological changes in the GM may not be noticed by 16S metataxonomy. To test this possibility, we studied the correlation of SCFA abundances in IBD patients and healthy subjects with the abundance of GM genes directly involved in SCFA metabolism. Our results show that CD patients exhibit a decrease in propionate that coincides with lower abundances in the terminal genes of the metabolic pathways leading to propionate production. When comparing these decreases with 16S information, we obtained a poor correlation, suggesting that geno-centric approaches such as the one developed here may be better suited for identifying the causal links between GM alterations and gut disease.

## 2. Materials and Methods

### 2.1. IBD Cohort Data

For this project, we studied a cohort of 132 IBD patients integrated in the second part of the Human Microbiome Project [15]; specifically, 38 patients diagnosed with UC, 67 with CD and 27 with no IBD (H). As stated in [16], none of these subjects had been diagnosed with known bleeding disorders, acute gastrointestinal infections, hepatitis, or immune-mediated diseases. We analyzed the publicly available multi-omic data from these subjects, allocated in the Inflammatory Bowel Disease Multiomics Database [16]. Specifically, we retrieved two separate tables with 546 metabolic (265 CD, 146 UC, and 135 H) and 178 metataxonomic (86 CD, 46 UC, and 46 H) merged profiles. We also downloaded the processed sequencing files of 1638 metagenomic stool samples (583 CD, 353 UC, and 362 H) from these patients, which were collected every two weeks, processed as detailed in [16] and sequenced on an Illumina HiSeq2500. Finally, we retrieved the metadata with the associations between the stool samples extracted in the original study and their corresponding patients.

### 2.2. Analysis of Metabolomic and Metataxonomic Data

Metabolic and metataxonomic profiles from the available samples and metadata with the diagnosed condition of the patients and their corresponding samples were parsed and analyzed through in-house-made Bash and R scripts. Plots were elaborated with the R package ggplot2 [17]. Statistical analysis was performed through pairwise Mann–Whitney tests with the Benjamini–Hochberg false discovery rate correction to assess the significance of the differences regarding the metabolic and 16S levels between the three groups of samples corresponding to the CD, UC, and H conditions.

### 2.3. Extraction of the GM Genes Involved in the Formation of Propionate

We defined the enzymes catalyzing the terminal reactions involved in the formation of microbial propionate through fermentative pathways, namely propionate kinase, propionate CoA transferase, and propionate CoA ligase. Individual reactions and their corresponding enzyme commission (EC) numbers were targeted through the metabolic-pathways databases MetaCyc, BRENDA, and KEGG [18,19,20]. After this, the EC numbers of target enzymes were linked to Pfam domains [21] by studying the associations established by ECDomainMiner [22].

The Unified Human Gastrointestinal Genome (UHGG) [14] is the most extensive database of sequenced GM genomes and microbial genes elaborated so far, and it was used in this study to retrieve the families of fermentative genes (FGs) coding for the enzymes involved in the formation of propionate. We specifically selected the 3205 pan-genomes of GM species with at least two characterized strains, which altogether contain more than 21 million genes. The profile hidden Markov model (pHMM) of every Pfam domain associated with a target EC number was queried with HMMER version 3.3(Howard Hughes Medical Institute, MD, USA) [23] against the pan-genomes for homologous sequences using the hmmsearch function, only allowing hits with an e-value less than 0.001 (-E 0.001). Genes retrieved multiple times with different Pfams associated to the same enzyme were deduplicated, and short sequences were eliminated from the resulting sets of genes to remove potential misannotations in the UHGG.

The resulting genes were concatenated to several protein-coding genes with experimentally validated enzymatic activity. The sequences of these genes were retrieved from MetaCyc, and they were used as controls to analyze their phylogenetic distance to the sets of genes defined previously. To do so, the genic groups were aligned with MAFFT version 7.271 [24] and represented in phylogenetic trees with IQ-TREE version 2.0.3 [25]. The trees were inspected to extract phylogenetically related sequences composing each family of FGs involved in the formation of propionate. As a result, we obtained four enzyme-coding FG clusters involved in the last steps of microbial propionate formation: *tdcD* and *pduW* (coding for the propionate kinase), *pct* (coding for the propionate CoA transferase), and *prpE* (coding for the propionate CoA ligase). A graphical representation of the complete workflow is provided in Figure S1.

### 2.4. Analysis of Gene Abundance in the Metagenomic Samples

We inspected the distribution of FG clusters in the metagenomic samples of the IBD cohort using DIAMOND version 2.0.2 [26]. Briefly, every metagenomic sample was aligned against each FG cluster, allowing only one alignment per sequencing read (–max-hsps 1) with an e-value less than 0.001 (–evalue 0.001) and a percentage of sequence identity between the read and each FG higher than 80% (–id 80). The sum of metagenomic reads aligned to each FG was considered an indicator of the genic abundance in each sample. The samples were posteriorly analyzed for differential abundance between the three conditions through a pairwise Mann–Whitney test with the Benjamini–Hochberg false discovery rate correction to assess the significance of the differences regarding the genic abundance between the three groups of samples corresponding to the CD, UC and H conditions.

## 3. Results

### 3.1. Propionate Is Depleted in Some Manifestations of IBD

**Figure 1:**
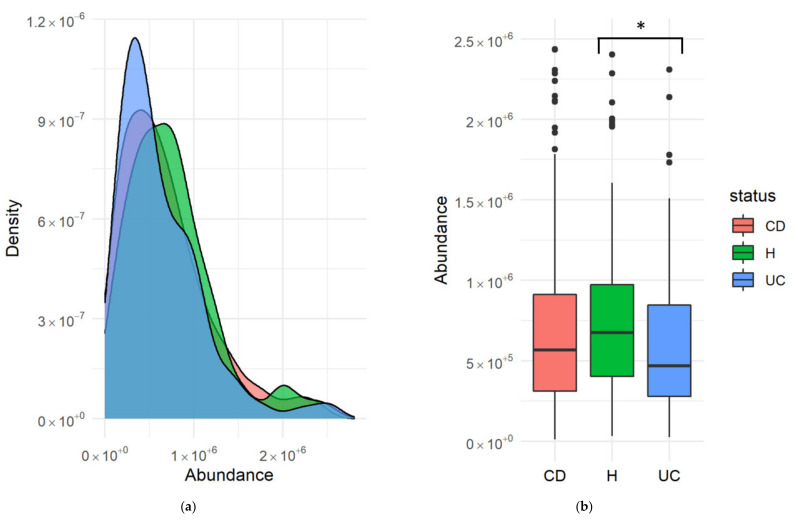
Levels of propionate in healthy subjects (H) and patients with Crohn´s disease (CD) and ulcerative colitis (UC) in absolute concentration units. (**a**) A kernel density estimate plot represents the abundance of propionate from the metabolomic profiles measured in fecal samples from the cohort analyzed in Lloyd-Price [19]. (**b**) A boxplot represents the abundance of propionate measured in fecal samples. Horizontal black bars represent median levels, while boxes and whiskers represent the data from first to third quartiles and from the quartiles to the minimum and maximum, respectively. Black dots correspond to outlier values outside the interquartile range. Statistical significance of the differences between groups was evaluated using a pairwise Mann–Whitney test with the Benjamini–Hochberg false discovery rate correction. No significant differences among groups were obtained for CD and H, while significant differences between H and UC were observed with *p* = 0.029 (*).

The abundance of SCFAs in healthy subjects, and CD and UC patients was obtained from the fecal samples of the IBDMDB cohort. Overall abundances were found to be not statistically significative for all groups and SCFAs analyzed, except propionate. Propionate levels were found to be significantly decreased in UC patients compared with healthy subjects (Figure 1). As shown in the figure, average levels were also lower in CD patients, although this difference was not found to be statistically significant.

### 3.2. Propionate Kinase Is the Most Abundant Terminal Enzyme Involved in the Formation of Microbial Propionate

**Figure 2:**
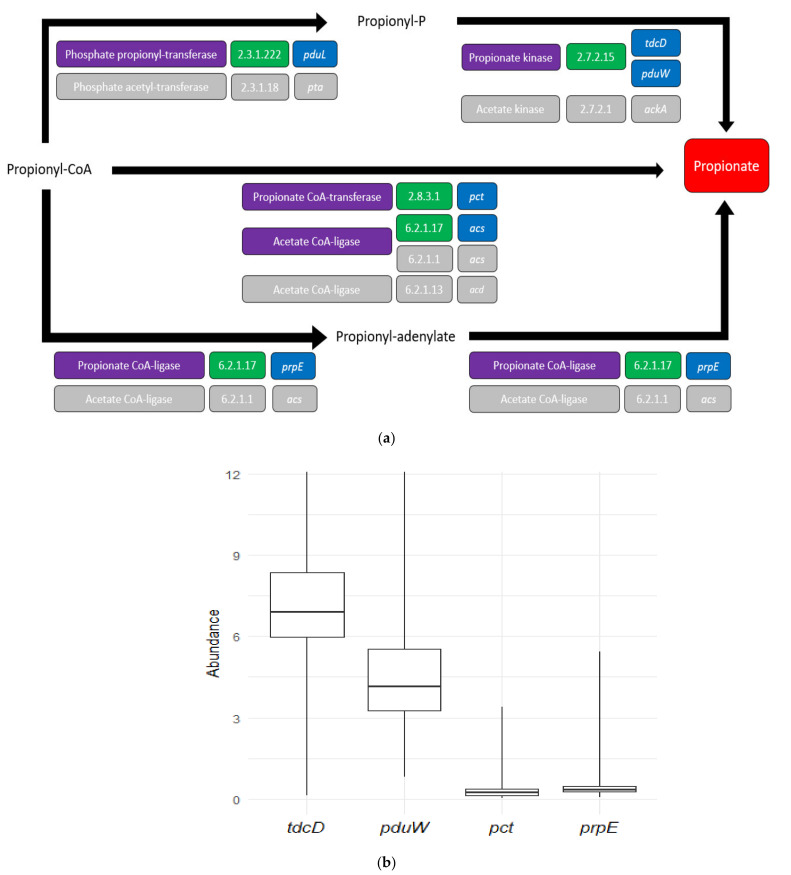
Characterization of the terminal genes involved in the formation of bacterial propionate. (**a**) Scheme with the three terminal reactions involved in the formation of propionate. Enzymes, EC numbers, and coding genes are represented in purple, green, and blue, respectively. As seen, some of the participating enzymes have substrate broadness between acetate and propionate (depicted in light grey). For example, the enzyme encoded by the *acs* gene forms acetate (EC 6.2.1.1) and propionate (EC 6.2.1.17); (**b**) boxplot representing the relative abundance of the four terminal genes involved in propionate formation in all the GM metagenomic samples. Horizontal black bars represent median levels, while boxes and whiskers represent the data from first to third quartiles and from the quartiles to the minimum and maximum, respectively. Abundances were measured as indicated in the Materials in Methods and are represented in thousands of reads.

Once propionate was identified as a potential SCFA altered in UC, we focused on the metabolic pathways leading to its formation. The enzymes catalyzing the terminal reactions involved in the production of propionate and the genes coding for these enzymes, were characterized through metabolic-pathways databases, as detailed in the Materials and Methods. As shown in Figure 2a, propionate can be formed through three different reactions. The first reaction is a dephosphorylation of propionyl-P that yields one ATP. This energetically favorable reaction is catalyzed by a propionate kinase (EC 2.7.2.15) that can be coded by two genes: *tdcD* and *pduW*. Propionate can also be formed through the transference of CoA cofactor in propionyl CoA to another metabolite through the enzyme propionate CoA transferase (EC 2.8.3.1) or acetate CoA ligase (coded respectively by *pct* and *acs*). This CoA-transferase route also conserves the energy of the CoA bond in the newly formed CoA-moiety of the co-substrate. Finally, propionate can be formed from propionyl-adenylate through a propionate CoA ligase (EC 6.2.1.17) encoded by the gene *prpE*. A complete diagram depicting propionate formation from pyruvate is provided in Figure S2.

The abundance of genes *tdcD* and *pduW* (coding for the propionate kinase), *pct* (coding for the propionate CoA transferase), and *prpE* (coding for the propionate CoA ligase) was measured in the metagenomic sequencing data from the stool samples of the IBD cohort, as described in the Materials and Methods. The results showed that the relative abundance of propionate kinase genes *tdcD* and *pduW* was higher than both CoA-mediating enzymatic genes (Figure 2b). This suggests that dephosphorylation is the major pathway employed by the GM to produce propionate.

### 3.3. Terminal Genes Involved in the Synthesis of Propionate Are Differentially Abundant in IBD

**Figure 3:**
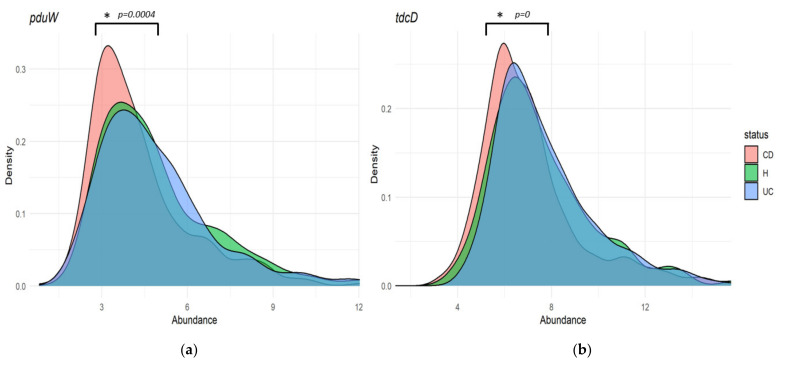

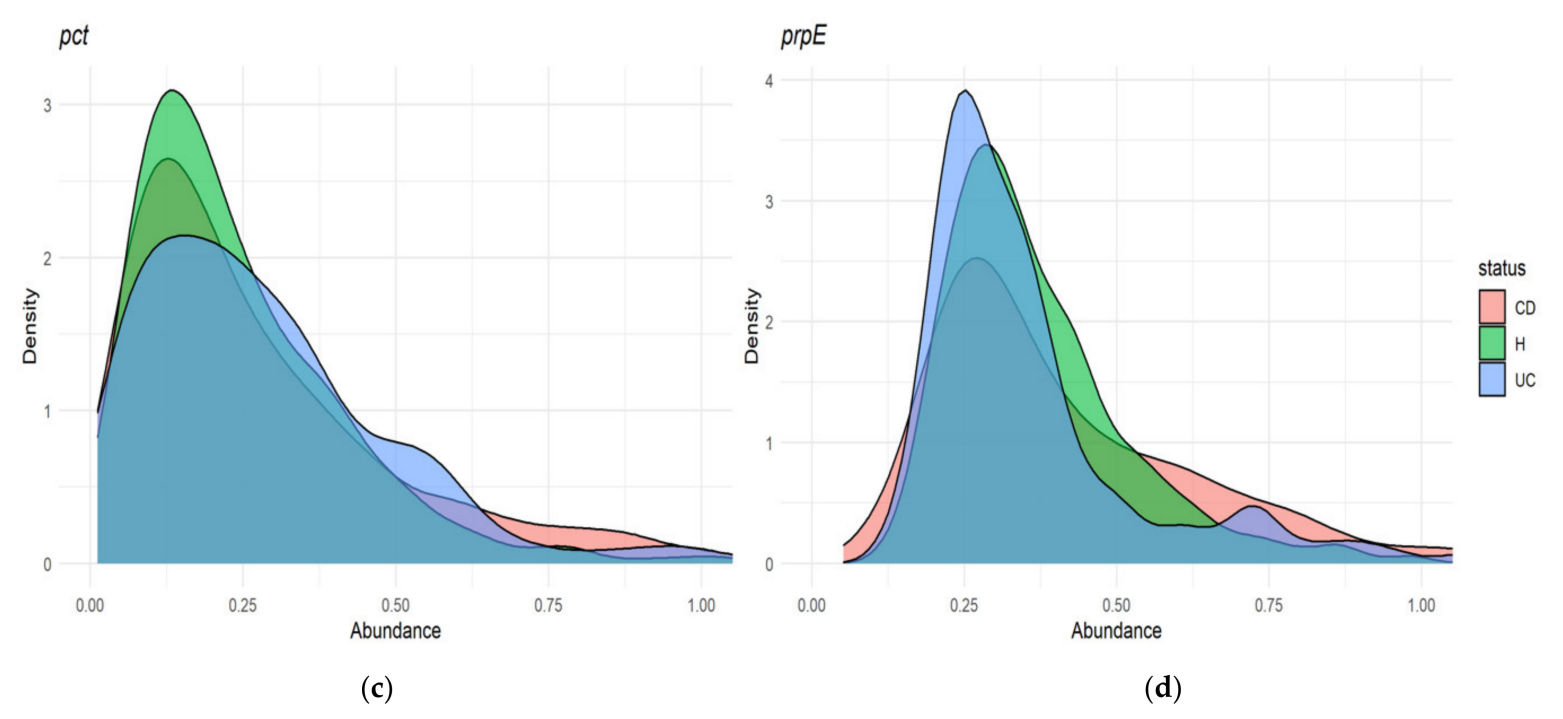
Abundance of genes involved in the terminal formation of propionate in healthy subjects (H) and patients with Crohn´s disease (CD) and ulcerative colitis (UC). (**a**,**b**) Propionate kinase genes *tdcD* and *pduW.* (**c**,**d**) Propionate CoA transferase gene *pct* and propionate CoA ligase gene *prpE*. Density plots represent the abundances of terminal microbial genes involved in the formation of propionate, from the metagenomic measures of the fecal samples from the cohort analyzed in [16]. Differences in abundances were evaluated using a pairwise Mann–Whitney test with the Benjamini–Hochberg false discovery rate correction. Significant differences among groups were obtained for *tdcD* and *pduW* between CD and H (*).

The abundance of genes *tdcD* and *pduW* (coding for the propionate kinase), *pct* (coding for the propionate CoA transferase), and *prpE* (coding for the propionate CoA ligase) was compared between the H, CD, and UC conditions. Pairwise Mann–Whitney tests with the Benjamini–Hochberg false discovery rate correction were used to assess the significance of the differences regarding the genic abundance between the three conditions. Abundances are presented as plots of their kernel density estimates. As shown in Figure 3a,b, propionate kinase coding genes *tdcD* and *pduW* are significantly more abundant in healthy samples when compared to CD (*p* = 0.0004 for *tdcD* and *p* = 0 for *pduW*, respectively). No significant differences among groups were obtained for UC and H. As shown in Figure 3c,d, no differences were found in *pct* or *prpE* either, probably due to their low relative abundance (Figure 2b).  Figures S3 and Figure S4 depict boxplots representing these differences.

### 3.4. Genes Involved in the Last Steps of Microbial Propionate Formation Have Taxonomic Shifts in IBD That Do Not Always Correlate with 16S Abundances

**Figure 4:**
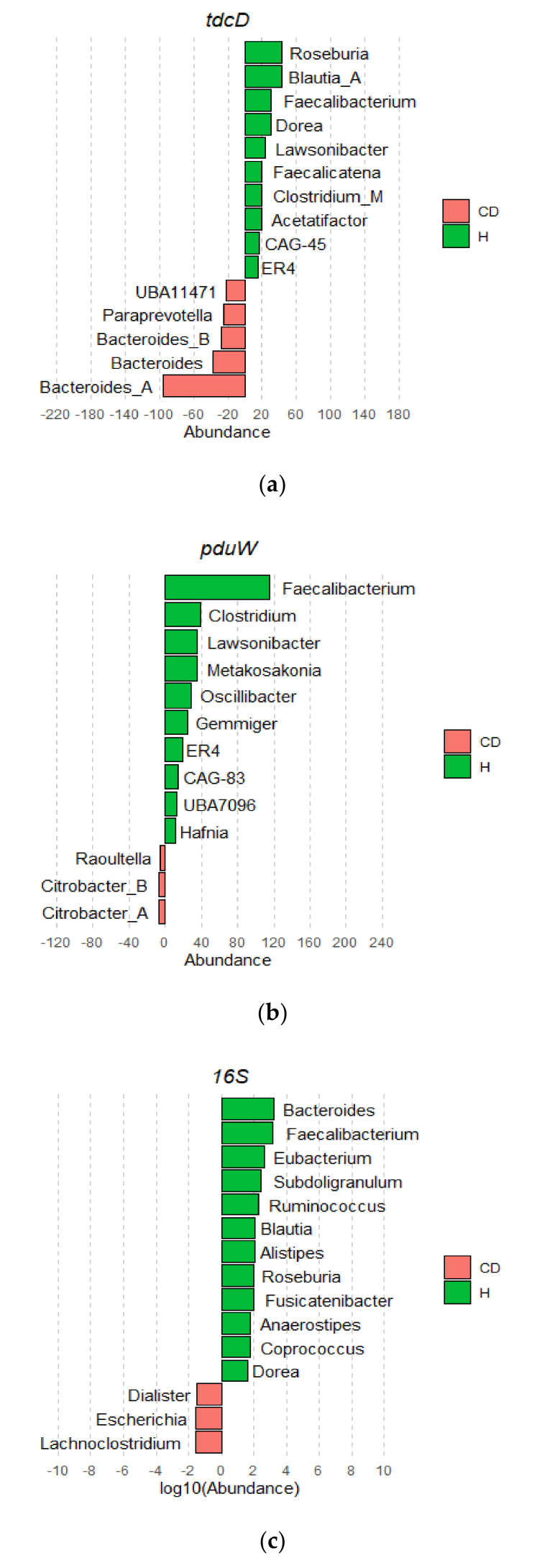
*tdcD, pduW,* and 16S average gene-abundance differences between healthy and CD conditions. (**a**,**b**) Differences in kinase abundances. Horizontal bars in the plots represent the difference in total average gene abundances between H and CD groups for *tdcD* and *pduW*. (**c**) Differences in average 16S abundance between the CD and H groups. Bars indicate the difference in log10 average abundances between H and CD groups, inferred from the 16S data in [16]. Only genera with the highest net variation (positive or negative) are represented.

The increases in *tdcD* and *pduW* abundances in the healthy condition compared with CD led us to try to determine whether this differential abundance was caused by changes in specific bacterial taxa. To do so, we calculated the average abundance of each kinase-coding gene in each genus and condition (UC, CD, and H). To compare these changes with absolute bacterial abundances, we retrieved the abundance of each genus in each condition in the same cohort of patients. This was achieved by plotting the 16S metataxonomic profiles obtained from fecal IBDMDB samples. Average gene levels were normalized, and the values for each genus were compared between H and CD patients (Figure 4a,b) and between H and UC patients (Figure S5. As shown in Figure 4a,b, H subjects showed an increased abundance of both *tdcD* and *pduW* kinase genes in multiple genus from class *Clostridia*. Specifically, kinase genes from members of family *Lachnospiraceae*, such as *Roseburia, Blautia,* or *Dorea* spp., as well as family *Ruminococcaceae* such as *Faecalibacterium* spp. were increased in the H condition. In contrast, kinase genes from *Bacteroidales* and, to a lesser extent, *Enterobacterales* were more abundant in CD patients.

**Figure 5:**
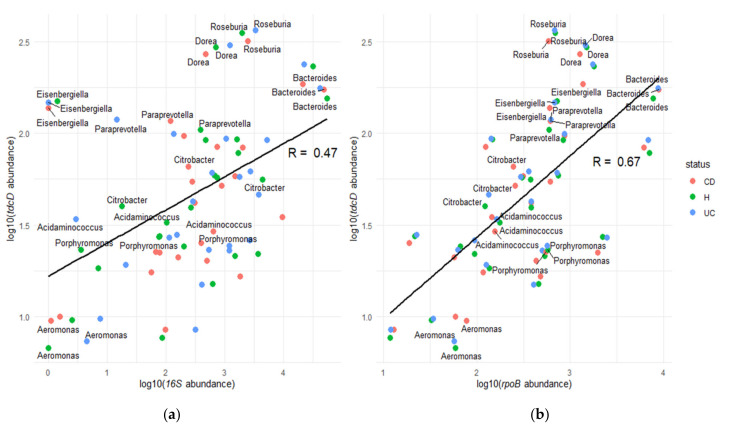
Dot plots between (**a**) *tdcD* and 16S, and (**b**) *tdcD* and *rpoB* average gene abundance. Dots in the plot represent the average gene abundance of GM bacterial genera in CD, UC, and healthy conditions on a log10 scale. Only genera with consistent taxonomic annotation between data from [14,16] are presented. Labels indicate bacterial taxa with higher differences between the 16S- and *rpoB*-based quantifications. The regression lines with the correlation coefficient between the corresponding genes are presented in both plots.

These results are in sharp contrast with 16S-derived abundances, which showed that H individuals had an overall increase in *Bacteroides* (Figure 4c). Changes in the abundance of propionate kinase genes thus correlated poorly with changes in taxon abundance, as determined by 16S. This discrepancy may be caused by *tdcD* not being core gene in many of the most significant taxa of the human GM (Table S1). The large genotypic variation observed for species in the GM [14] means that many biochemical pathways are present only in a fraction of the strains of a given species. Thus, changes in *tdcD* abundance may be shifts in certain strains rather than in particular species. However, the discrepancy between *tdcD* and 16S abundances may also be attributed to a methodological artifact. Although *tdcD* is quantified directly from metagenomic data, 16S counts are retrieved from metataxonomic analysis, which imply significant differences in DNA amplification and sequencing. To check whether this discrepancy between the abundances of *tdcD* and 16S counts could be ascribed merely to technical reasons, we measured the overall levels of the core gene *rpoB* in the most significant taxa. *rpoB* codes for the beta subunit of the RNA polymerase, and it was quantified in the metagenomic data from the stool samples, as described in the Materials and Methods. By comparing *tdcD* and *rpoB* abundances, we could thus rule out discrepancies due to different methodological procedures. As presented in Figure 5, *rpoB* and *tdcD* abundances showed a poor correlation, indicating that overall bacterial abundance cannot be used as a proxy to estimate *tdcD* abundance.

## 4. Discussion

Inflammatory diseases like IBD are complex, multi-trait disorders in which causal links are difficult to identify. There is evidence pointing to the involvement of the GM in development of IBD, and the role of SCFAs in homeostasis and immunomodulation in the gut has been indicated as a possible cause. However, comprehensive multi-omic studies have failed to univocally ascribe a role for the GM in IBD [13,16]. A major problem arising is the lack of consistency in the relative abundances of different taxa, which appear uncorrelated in healthy and IBD-affected patients in different studies. One possible reason for this discrepancy is the large genomic diversity within the bacterial species in the GM [14]. Since species may hide large genomic variations, alterations in the GM may be due to changes in genes rather than changes in species. From this perspective, we re-analyzed multi-omic results for an IBD cohort [16], trying to link changes in SCFA abundance with differential abundances in the genes responsible for the metabolization of these genes. As shown in Figure 1, propionate was found to be significantly decreased in UC patients compared with healthy subjects. It was also decreased in CD patients, although not significantly.

Propionate is produced by a variety of GM species through several different pathways, including lactate fermentation; succinate degradation; the degradative pathways of certain amino acids, such as alanine and threonine; and the fermentation of pyruvate to propanoate through the succinate and acrylate pathways, among others [5]. Analyzing the variety of enzymes involved in these and other relevant pathways would be daunting, but by focusing on the common terminal reactions that directly lead to propionate, we were able to narrow our search. There are two known terminal reactions leading to propionate (Figure 2a). It can be produced from propionyl CoA by propionate CoA transferases and ligases, such as in the acrylate pathway, succinate/propionate conversion, pyruvate and lactate fermentations, and alanine degradation. Alternatively, it can be produced from propionyl phosphate via the reverse activity of propionate kinases, such as in the threonine degradation pathway and the methylcictrate cycle. Our results indicated that, from these, propionate kinases are more abundant in the GM metagenome (Figure 2b). This is a surprising finding, as propionyl-CoA-mediated reactions are much more frequent in fermentative and degradative pathways, and even routes that have a kinase in their final steps (threonine degradation and the methylcitrate pathway) also include a propionyl CoA intermediary. The higher abundance of *tdcD* and *pduW* is thus puzzling, yet it is known that SCFAs, CoA ligases, and transferases show extensive substrate promiscuity. Acetate CoA enzymes can metabolize acetate and propionate in multiple situations [5], as shown by some GM genera such as *Phascholarctobacterium* [27]. Further research is thus required to analyze the extent to which substrate promiscuity plays a role in SCFA production in the GM.

Regarding their relative abundances in H, CD, and UC groups, *prpE* was decreased in UC patients (Figure 3d). This result correlates with the reduction in propionate levels in this IBD manifestation. Propionate kinases were found to be significantly under-represented in CD patients (Figure 3a,b). By ascribing each propionate kinase count to its cognate species, we were able to obtain taxon-specific abundances. These abundances showed that CD patients have an under-representation of counts from *Faecalibacterium*, *Roseburia*, *Blautia,* and *Clostridium*, which coincides with an increased abundance of these genera in healthy subjects, according to 16S (Figure 4c). Previous studies have reported decreased levels of these genera in CD, many of which are known SCFA producers [28,29]. *R. hominis,* for example, was shown to promote gut barrier function and immunity in murine and in-vitro models [30] and it was associated with a protective role against IBD [27]. Similarly, *Faecalibacterium prausnitzii,* one of the most abundant bacteria in GM, was proposed as a potential biomarker given its depletion in CD and UC patients [29]. Our results showed that *tdcD* from *Roseburia* and *tdcD* and *pduW* from *Faecalibacterium* are increased in healthy samples (Figure 4a,b). This implies that the protective role of these taxa is due to the production of propionate through the kinases encoded by these genes.

In other taxa, however, comparing taxon-specific counts and 16S-derived abundances yielded conflicting results (Figure 4). The most obvious discrepancy occurred in *Bacteroides*, which showed a decrease in CD patients, yet *tdcD* genes from *Bacteroides* were increased. This discrepancy may arise from the genomic plasticity associated with many GM species [14]. As metabolic genes are not part of the core genome in many taxa (Table S1), shifts in the strain composition of the GM may alter its metabolic capabilities without reflecting in the 16S profile. This may be observed when total abundances and gene-specific abundances are compared (Figure 5). The lack of correlation between these two indicators strongly suggests that changes in strain composition are also a key player in alterations of the GM. This also means that 16S abundances probably yield a skewed view of GM changes. Geno-centric approaches, such as the one developed here, may help to better understand GM alterations associated with pathological conditions. Characterizing the genomic diversity of the GM is thus fundamental to understanding the metabolic activity encoded in the core and accessory genomes of individual species. This will contribute to refining the genic target that constitutes the basis of therapeutic strategies, such as stool transplant protocols, which are developed only from a taxonomic point of view. By linking metabolites and genomic abundances, they may also help us understand the causal links, if any, between changes in the microbiome and the onset and evolution of IBD and other diseases.

## Data Availability

The datasets analyzed for this study can be found in the following repositories: UHGG is allocated at http://ftp.ebi.ac.uk/pub/databases/metagenomics/ (accessed date on 5 January 2021); IBD cohort data is allocated at https://ibdmdb.org/ (accessed date on 5 January 2021). Analysis scripts elaborated are available at https://github.com/JuanmaMedina/GM_IBD/ (accessed date on 5 January 2021).

## Supplementary Materials

The following are available online at https://www.mdpi.com/article/10.3390/jcm10102176/s1, Figure S1: Graphical representation of the computational workflow, Figure S2: Propionate formation from pyruvate, Figure S3: Boxplots showing abundance of propionate kinase genes *tdcD* and *pduW*, Figure S4: Boxplots showing abundance of propionate CoA transferase gene pct and propionate CoA ligase gene *prpE*, Figure S5: Differences in *tdcD* and *pduW* abundances between H and UC , Table S1: Presence of *tdcD* gene in the strains of the most representative GM species.
