# Supplementary figures and images for: Propionate Fermentative Genes of the Gut Microbiome Decrease in Inflammatory Bowel Disease
Source: J Clin Med. 2021 May 18;10(10):2176. doi: 10.3390/jcm10102176 (PMC8157882; doi:10.3390/jcm10102176)

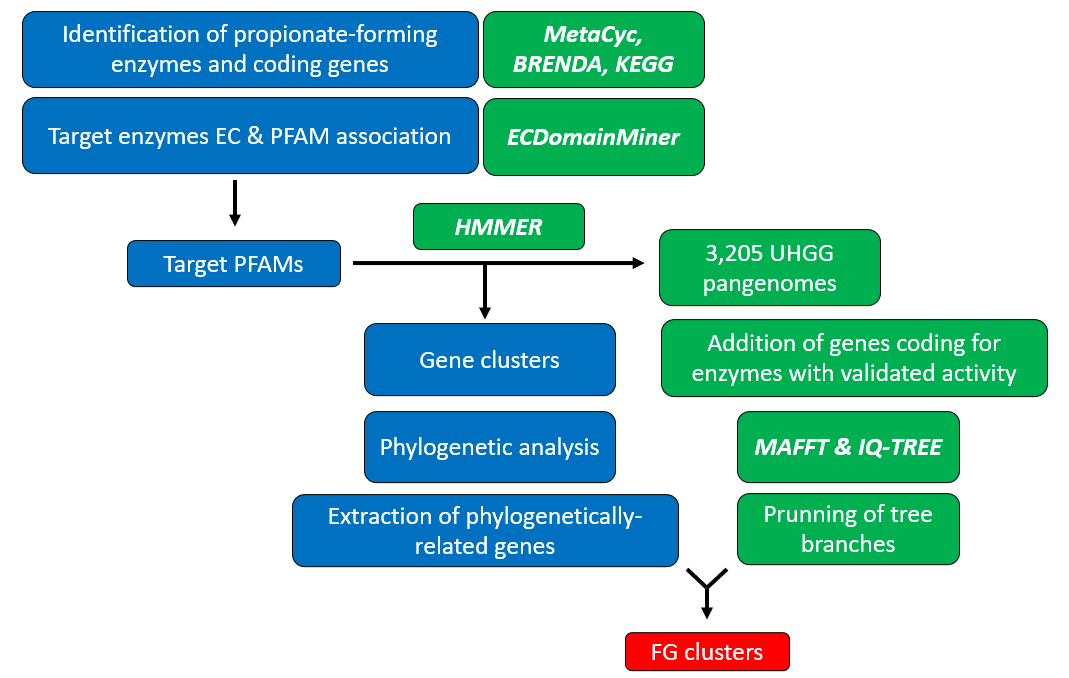

Supplement: Supplementary file 1 [file jcm-10-02176-s001.zip › jcm-1192097- supplementary/Supplementary_Figure_1.png]

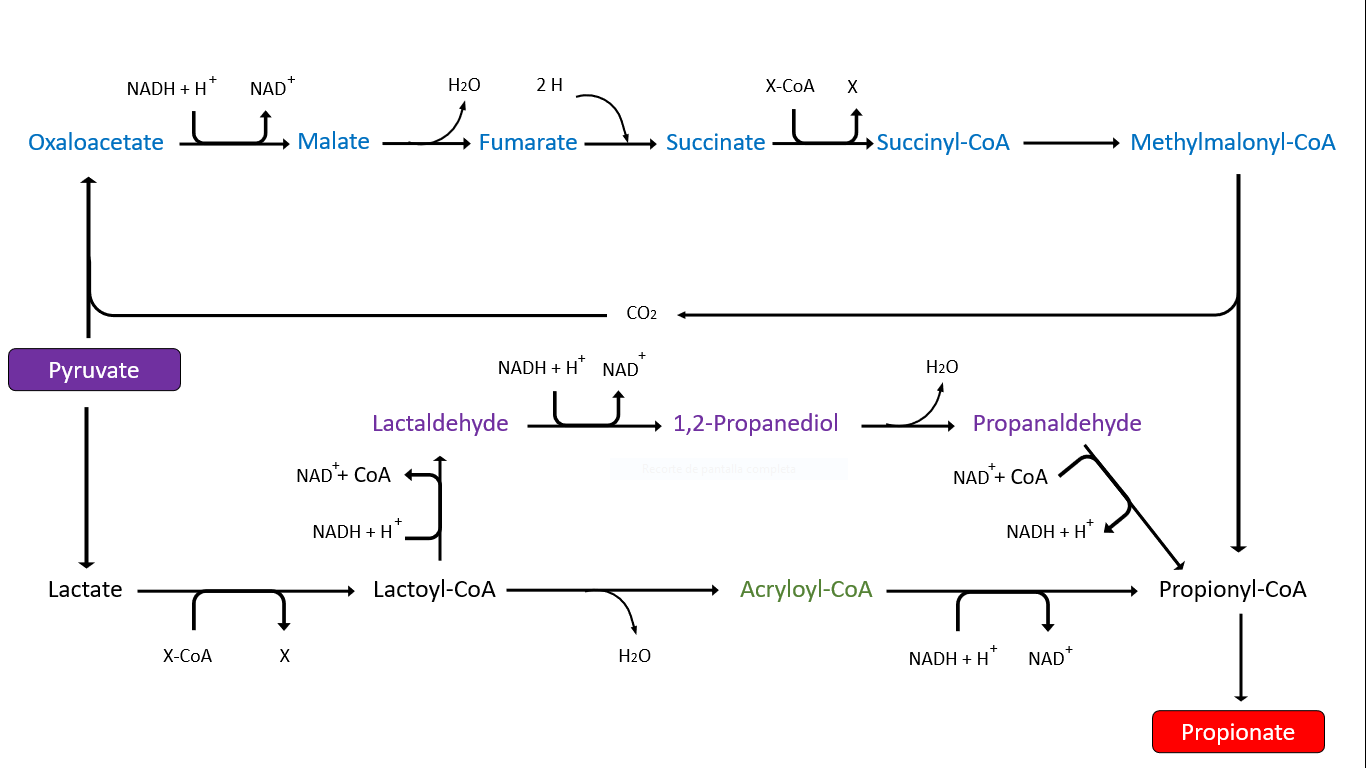

Supplement: Supplementary file 1 [file jcm-10-02176-s001.zip › jcm-1192097- supplementary/Supplementary_Figure_2.png]

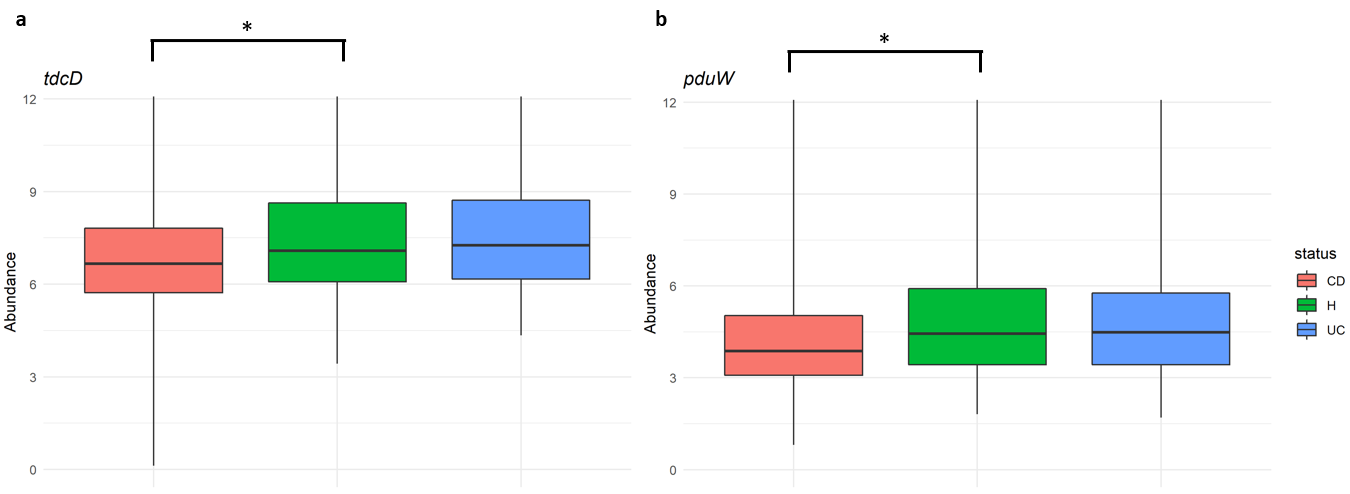

Supplement: Supplementary file 1 [file jcm-10-02176-s001.zip › jcm-1192097- supplementary/Supplementary_Figure_3 (2).png]

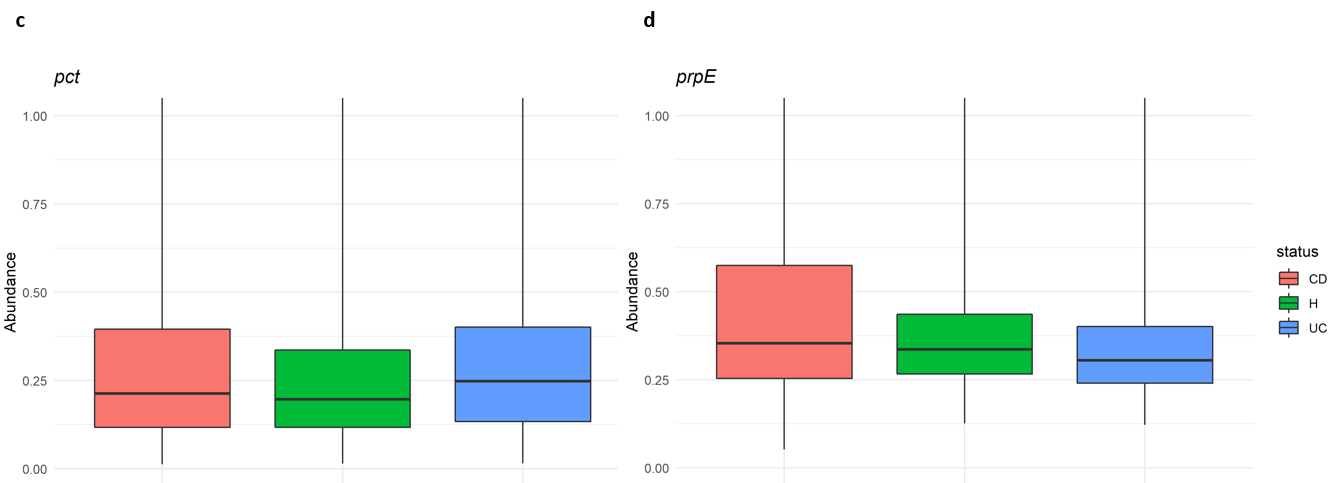

Supplement: Supplementary file 1 [file jcm-10-02176-s001.zip › jcm-1192097- supplementary/Supplementary_Figure_4.PNG]

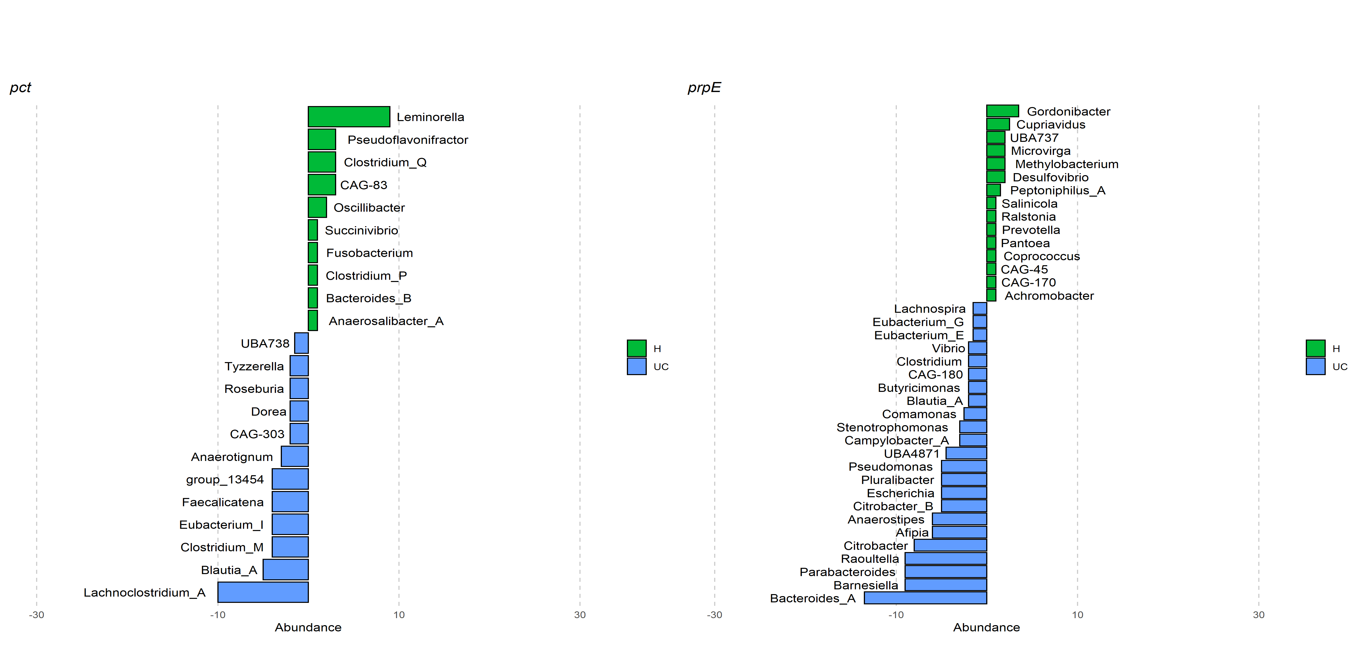

Supplement: Supplementary file 1 [file jcm-10-02176-s001.zip › jcm-1192097- supplementary/Supplementary_Figure_5 (2).png]
